# Piezo inkjet formation of Ag nanoparticles from microdots arrays for surface plasmonic resonance

**DOI:** 10.1038/s41598-024-55188-1

**Published:** 2024-02-27

**Authors:** Brahim Aïssa, Adnan Ali

**Affiliations:** 1 Qatar Environment and Energy Research Institute (QEERI), Hamad Bin Khalifa University (HBKU), Qatar Foundation, P.O. Box 34110, Doha, Qatar; 2 Department of Chemical Engineering, Jeju National University, Jeju, 63243 Korea

**Keywords:** Piezo-inkjet printing, Nanoparticle, Plasmon resonance, Morphological properties, UV–Vis analysis, Engineering, Materials science, Nanoscience and technology, Optics and photonics, Physics

## Abstract

The study aims to explore a novel approach for fabricating plasmonic nanostructures to enhance the optical properties and performance of various optoelectronic devices. The research begins by employing a piezo-inkjet printing technique to deposit drops containing Ag nanoparticles (NPs) onto a glass substrate at a predefined equidistance, with the goal of obtaining arrays of Ag microdots (Ag-µdots) on the glass substrate. This process is followed by a thermal annealing treatment. The printing parameters are first optimized to achieve uniform deposition of different sizes of Ag-µdots arrays by controlling the number of Ag ink drops. Subsequently, the printed arrays undergo thermal annealing at various temperatures in air for 60 min, enabling precise and uniform control over nanoparticle formation. The printed Ag nanoparticles are characterized using field emission scanning electron microscopy and atomic force microscopy to analyze their morphological features, ensuring their suitability for plasmonic applications. UV–Vis spectrophotometry is employed to investigate the enhanced surface-plasmonic-resonance properties of the printed AgNPs. Measurements confirm that the equidistant arrays of AgNPs obtained from annealing Ag microdots exhibit enhanced light-matter interaction, leading to a surface plasmon resonance response dependent on the Ag NPs’ specific surface area. These enhanced surface plasmonic resonances open avenues for developing cutting-edge optoelectronic devices that leverage the benefits of plasmonic nanostructures, thereby enabling new opportunities for future technological developments across various fields.

## Introduction

The trend towards miniaturization in the electronics industry stands as a pivotal driver for the demand for integrated devices (IDs). As electronic devices continue to shrink and become more compact, there is a growing need for components that can seamlessly fit into these reduced form factors without compromising performance. Printed Electronics (PEs) offers a solution to this challenge and is a rapidly emerging and innovative technology for the fabrication of IDs^1^. When combined with additive manufacturing, PEs provide a versatile platform for prototyping and fabricating next-generation microelectronic devices. These devices encompass a wide array of applications, including displays, IoT sensors, biomedical devices, batteries, solar cells, MEMS devices, and wearable electronics^2–8^. Recent years have witnessed significant progress in printing techniques, particularly in the fabrication of 2D planar structures and free-standing 3D architectures^9–12^. Notably intriguing in this context are the methods of direct writing that enable the generation of printed features in the micrometer range without the use of an electric field, eliminating potential risks to fragile electronic components on the substrate^13,14^.

Inkjet printing is a non-impact direct printing technology that deposits ink in a patterned array known as the dot matrix. It operates on the principle of digitally controlled ejection of fluid drops from a small aperture to a pre-specified position^15,16^. The concept of inkjet printing can be traced back to Lord Rayleigh in 1878, who proposed a liquid jet with a constant radius capable of falling vertically under gravity^17^. Piezoelectric inkjet printing is a widely utilized fluid-dispensing technique that enables fully digital-driven processing^18^. Typically, the fluid viscosity and surface tension for piezoelectric print heads should fall within the ranges of below 40 cP and 20–70 mN m^−1^, respectively^19^. When a sufficient force is applied to the orifice, the fluid is extruded, forming microdroplets with a diameter usually 1.2–2 times that of the orifice^19–21^. Drop-on-demand (DOD) piezo inkjet printing offers a cost- and time-effective additive process for mask-less micropatterning. Piezo-Inkjet printing is a promising Drop-on-Demand (DOD) technology that enables the patterning of materials with minimal waste. Moreover, it is user-friendly and yields high-resolution printing results, providing greater control over the shape and size of the ink release. Additionally, it has outperformed the spin coating technique as an effective manufacturing method for fabricating organic or polymer light-emitting devices^22–24^. In fact, ongoing research activities are exploring the inkjet printing of high-efficiency solar cells using inorganic materials^21,24^.

According to previous research^21,24^, the global printed electronics market size is projected to exceed US$ 28.07 billion by 2030, expanding at a growth rate of 18.5% until that year. The advancement of this application field depends on the effective integration of dielectric, semiconductor, and conductive materials over large, flexible substrates, resulting in thin, lightweight, flexible, and reliable devices with reduced costs and material waste^25^.

On the other hand, solid-state dewetting (SSD) describes the transformation of a thin film into an energetically favored set of droplets and/or particles. Interestingly, SSD characterizes this process as occurring at temperatures well below the melting temperature of the bulk material^21,26–28^. In practical terms, SSD serves as a degradation mechanism in the application of thin films for electronic, magnetic, and optical purposes, imposing an upper limit on the thermal exposure of devices. However, the efficiency and simplicity of the SSD process have been increasing, positioning it as an alternative fabrication route for nanoparticle (NP) arrays with controlled shape, spacing, periodicity, and composition^25,26^. Applications of SSD span from magnetic storage arrays^29,30^ to plasmonic systems^31,32^. Despite significant advancements in the SSD topic, the mechanisms involved in NP formation are still under discussion. While it was generally accepted, based on the pioneering works of Brandon et al.^33^, Presland et al.^34^, and Srolovitz et al.^35,36^, that SSD is governed by surface self-diffusion, recent research by Kovalenko et al.^37^, Amram et al.^38^, and Kosinova et al.^39,40^ has shown that grain boundary and interface diffusion can also play crucial roles.

The solid-state dewetting (SSD) of metallic thin films is widely acknowledged as an effective method for producing customized micro- and nanostructures with diverse potential applications, including plasmonics^39,41–44^. In metal nanoparticles (NPs), the conduction band and valence band are closely situated, allowing electrons to move freely. These free electrons contribute to the emergence of a surface plasmon resonance (SPR) absorption band^45,46^. For instance, silver (Ag) NPs exhibit high efficiency in absorbing and scattering light. This strong interaction with light occurs because the conduction electrons on the metal surface collectively oscillate when excited by light at specific wavelengths^47–50^.

Research indicates that the physical, optical, and catalytic properties of AgNPs are significantly influenced by their size, distribution, shape, and surface properties^51^. In the case of small metal particles (i.e., diameter < 20 nm), absorption spectra depend solely on dipole oscillation. As the NP size decreases, the SPR peak shifts towards shorter wavelengths, and as the size decreases further, absorption spectra become weak and broad^52,53^.

In this study, diverse arrays of silver nanoparticle (AgNP) clusters are generated from arrays of Ag microdots (Ag-µdots) using a piezo inkjet printer 2850 through solid-state dewetting (SSD) at elevated temperatures. For this purpose, varying amounts of Ag drops (ranging from one to five drops of the same volume) are deposited. We examined the impact of the number of drops on the aspect ratio of Ag-µdots. Similarly, we explored the influence of temperature on the dewetting of Ag-µdots and the formation of AgNPs, with the annealing duration held constant at 1 h. Furthermore, we investigated the correlation between the SSD temperature, the specific surface area of the formed nanoparticles (NPs), and their optical properties for two different arrays.

## Fabrication method

### Piezo inkjet printing

AgNP-based ink is procured from Advanced Nano Products (ANP). The ink, containing 30–35 wt% AgNPs, is dispersed in Triethylene Glycol Monoethyl Ether (TGME) with a viscosity of 10–17 cPs and a surface tension of 35–38 dyn.cm-1. The ink cartridge specifications include 16 nozzles with a 21 μm nozzle diameter positioned at 254 μm spacing, and a 1 pL calibrated drop size. The maximum jetting frequency is 20 kHz. Both the printing cartridge temperature and substrate temperature are maintained at 20 °C, with the jetting frequency set at 20 kHz. The applied voltage to the piezoelectric inkjet head is 25 V, and the distance between the substrate and nozzle orifice is set at 1200 µm. Only one nozzle out of sixteen is utilized.

The spatial resolution of inkjet printing can be adjusted by varying the cartridge-mounting angle, which, in turn, increases the drop spacing. In this study, the drop spacing is maintained at 50 μm. To control the drop size and ejection from the nozzle, the single-pulse voltage waveform has been optimized for the ink to restrict the volume of the drop, enabling the printing of Ag microdot patterns with a 50 µm drop spacing. For Ag-µdots printing, the ink reservoir is depressed by a bias voltage (i.e., 0 V), causing the piezoelectric element (PZT) to move back to the relaxed position. The ink is pulled into the reservoir, and then the chamber is compressed, generating pressure to eject the drop.

**Figure 1:**
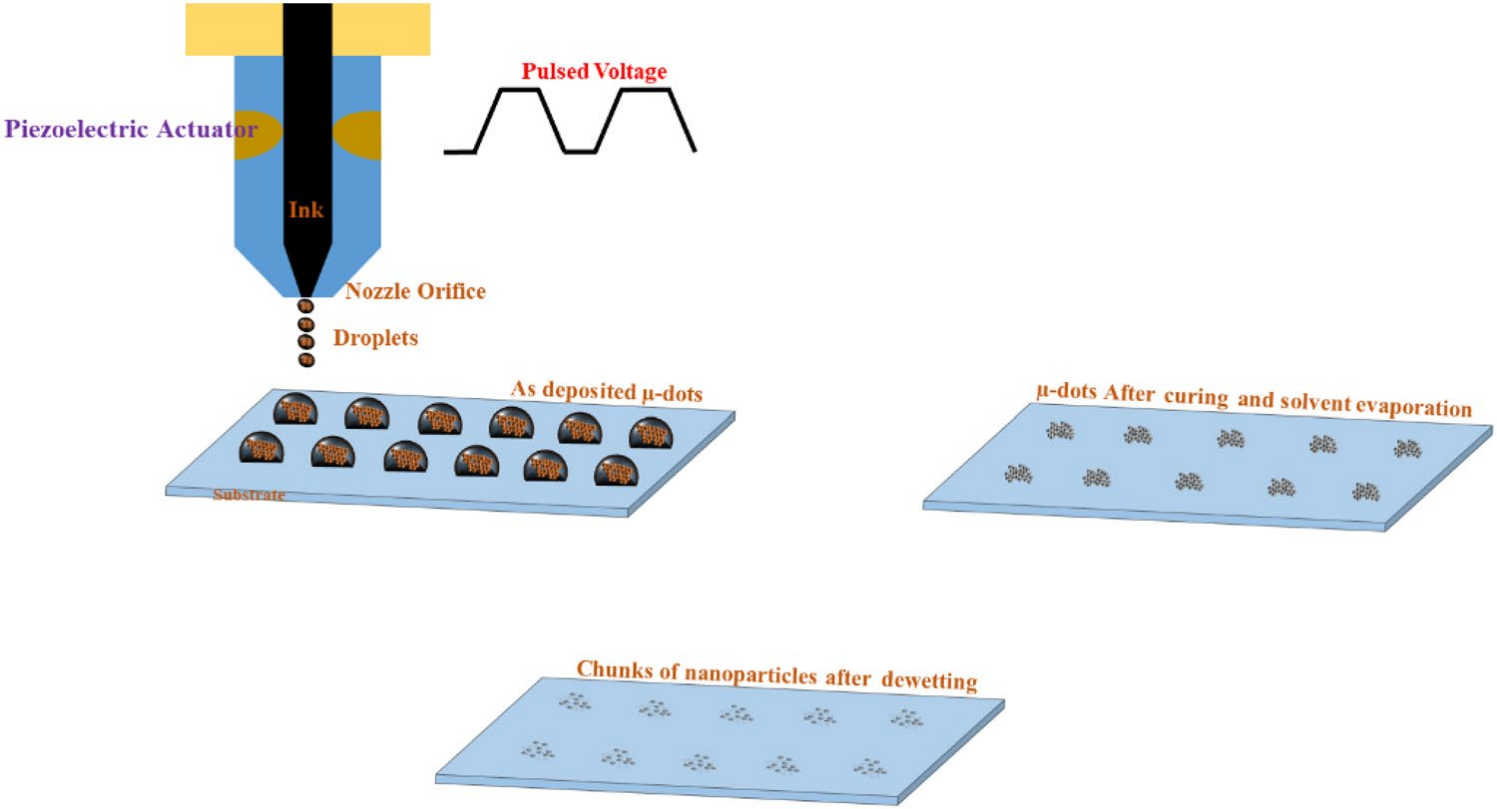
Schematic showing the metallic nanoparticles microdots fabricated by the Piezo Inkjet Printing process.

A borosilicate glass substrate (Corning 1737F) is used, meticulously cleaned with acetylacetone, rinsed with ethanol, and dried with nitrogen gas. After printing, the arrays of Ag ink drops on the glass substrate are cured at 150 °C on a hot plate for 30 min to remove solvents. In Fig. 1, a schematic of the printed equidistant drops by the piezo inkjet printer containing metallic NPs is illustrated.

Morphological characterizations of the printed Ag microdots (Ag-µdots) were performed using Scanning Electron Microscopy (SEM) equipped with Energy Dispersive Spectroscopy (EDS) (Jeol-JSM-6300F at 15 kV accelerating voltage with a 2 nm sputtered Au conductive film). Atomic Force Microscopy (NanoScope III, Digital Instrument), operated in contact mode at room temperature in ambient air, was also employed.

**Figure 2:**
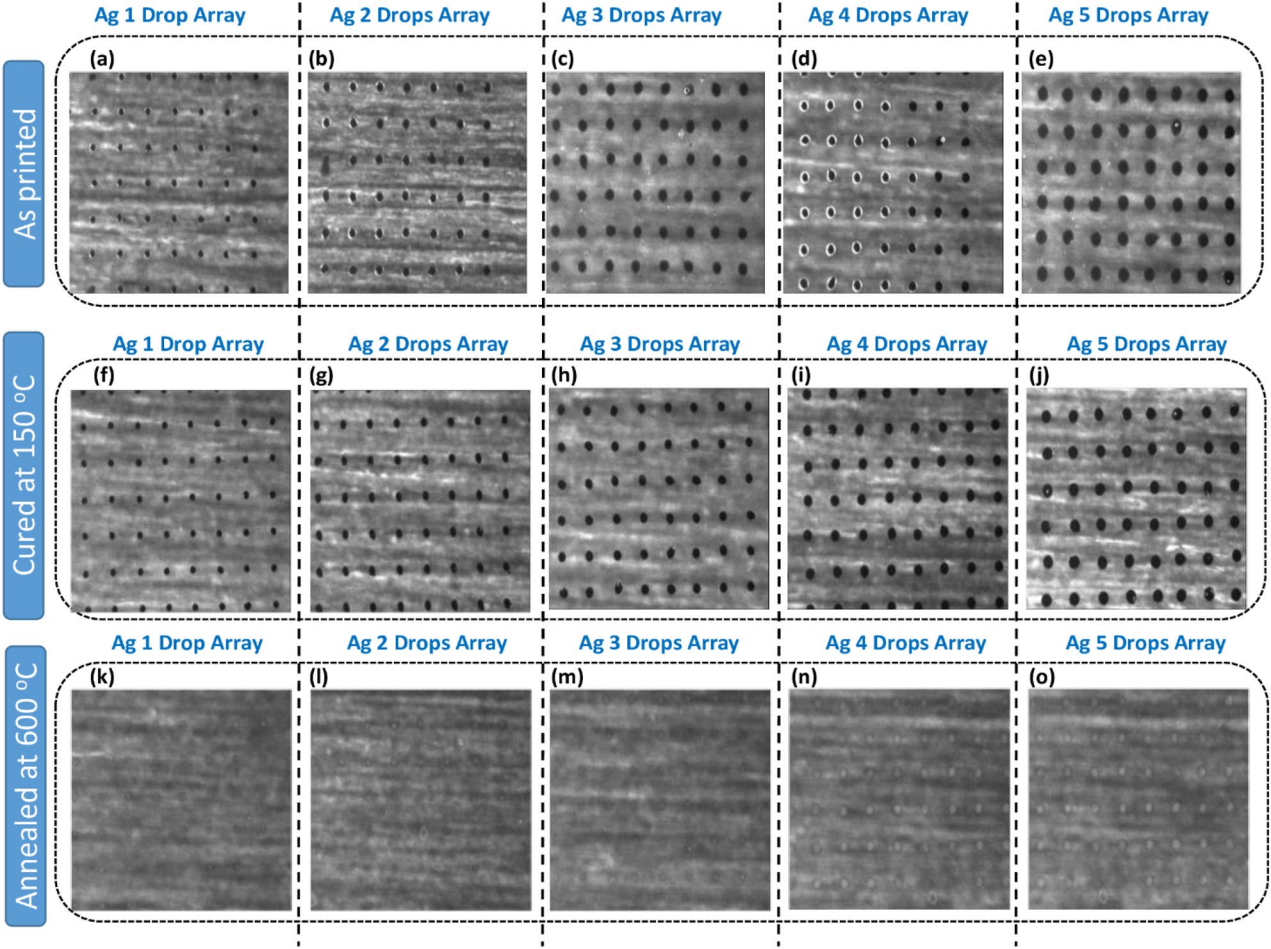
Optical microscopy of (1–5) drops Array of Ag µ-dots: (**a**–**e**) as-deposited, (**f**–**j**) Cured at 150 °C for 30 min, and (**k**–**o**) dewetted at 600 °C for 1 h.

In Fig. 2, arrays of Ag drops ranging from 1 to 5 drops, captured by the in-built camera of the piezo inkjet printer, are displayed. Figure 2a–e illustrate the as-deposited drops immediately after printing on the substrate. The increase in size is evident when moving from left to right (i.e., from 1 to 5 drops deposited at the same position). Similarly, images of the Ag drops were captured after curing at 150 °C and annealing at 600 °C, as shown in Fig. 2f–j and k–o, respectively.

## Results and discussion

**Figure 3:**
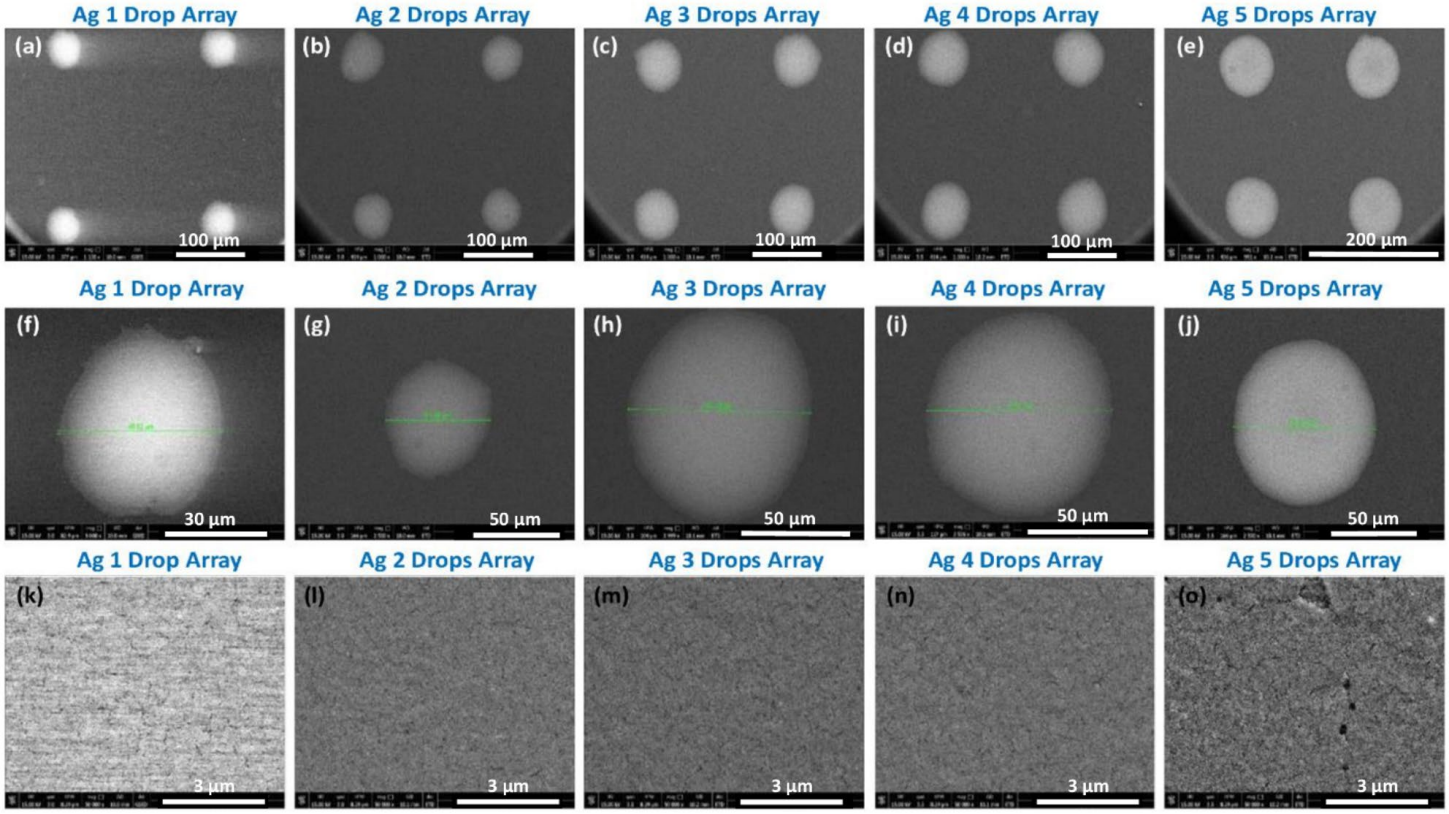
Typical FESEM images of (1–5) drops Array of Ag µ-dots from low (**a**–**e**) to high (**f**–**o**) magnifications of the Ag µ-dots cured at 150 °C for 30 min.

Figure 3 displays typical Field Emission Scanning Electron Microscopy (FESEM) images at varying magnifications (low: a-e, high: f-o) of the Ag microdots (Ag µ-dots) cured at 150 °C for 30 min. The Energy Dispersive Spectroscopy (EDS) analysis of these Ag µ-dots cured at 150 °C is presented in Figure S1(A). A prominent Ag peak around 3 keV in all Ag 1 to 5 µ-dots confirms the presence of silver. Other observed peaks are attributed to the glass substrate, and the Au peak results from the sputtered thin film used for FESEM analysis.

**Figure 4:**
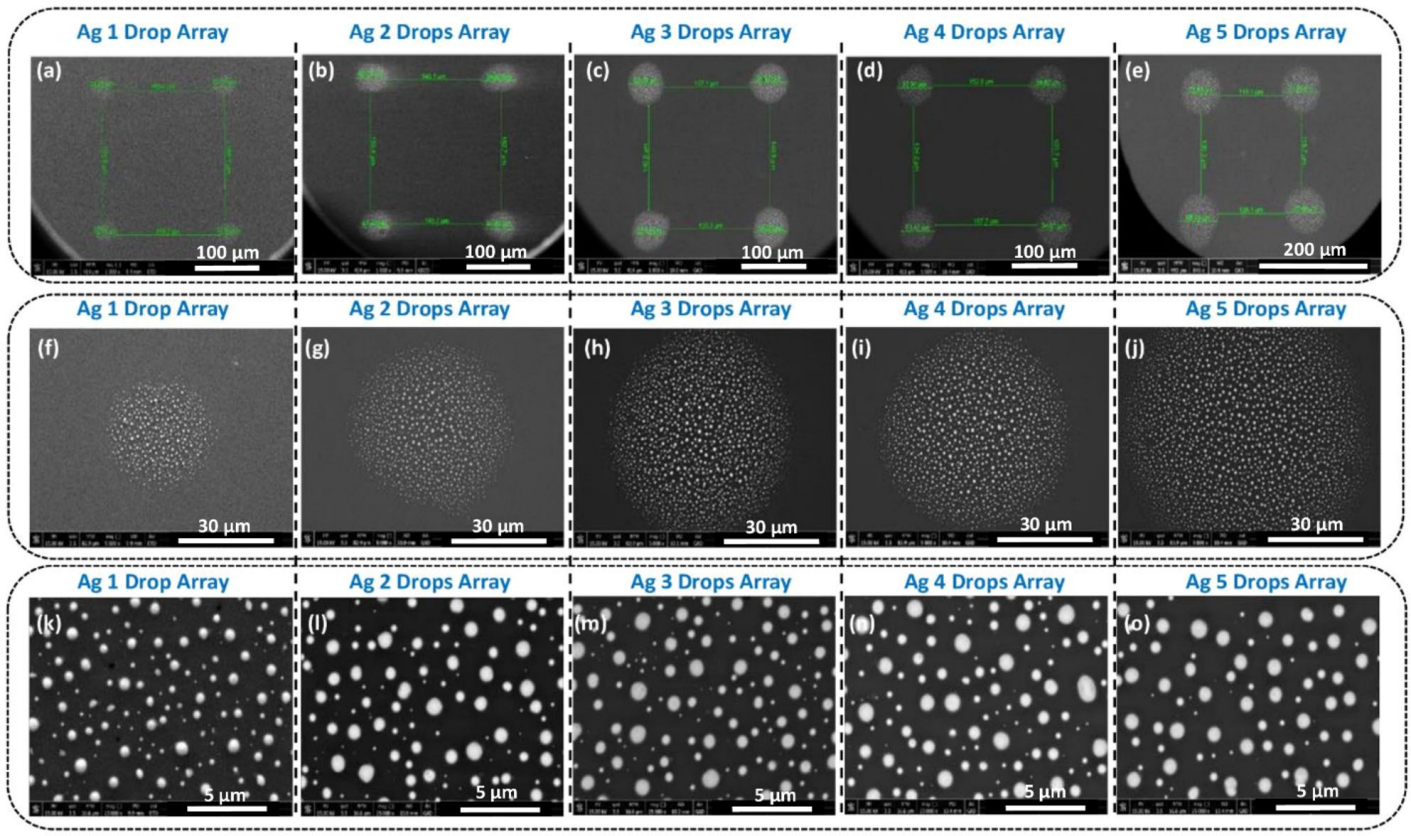
FESEM images of low (**a**–**e**) to high magnifications of (1–5) drops Array of Ag µ-dots cured at 600 °C for 60 min.

**Figure 5:**
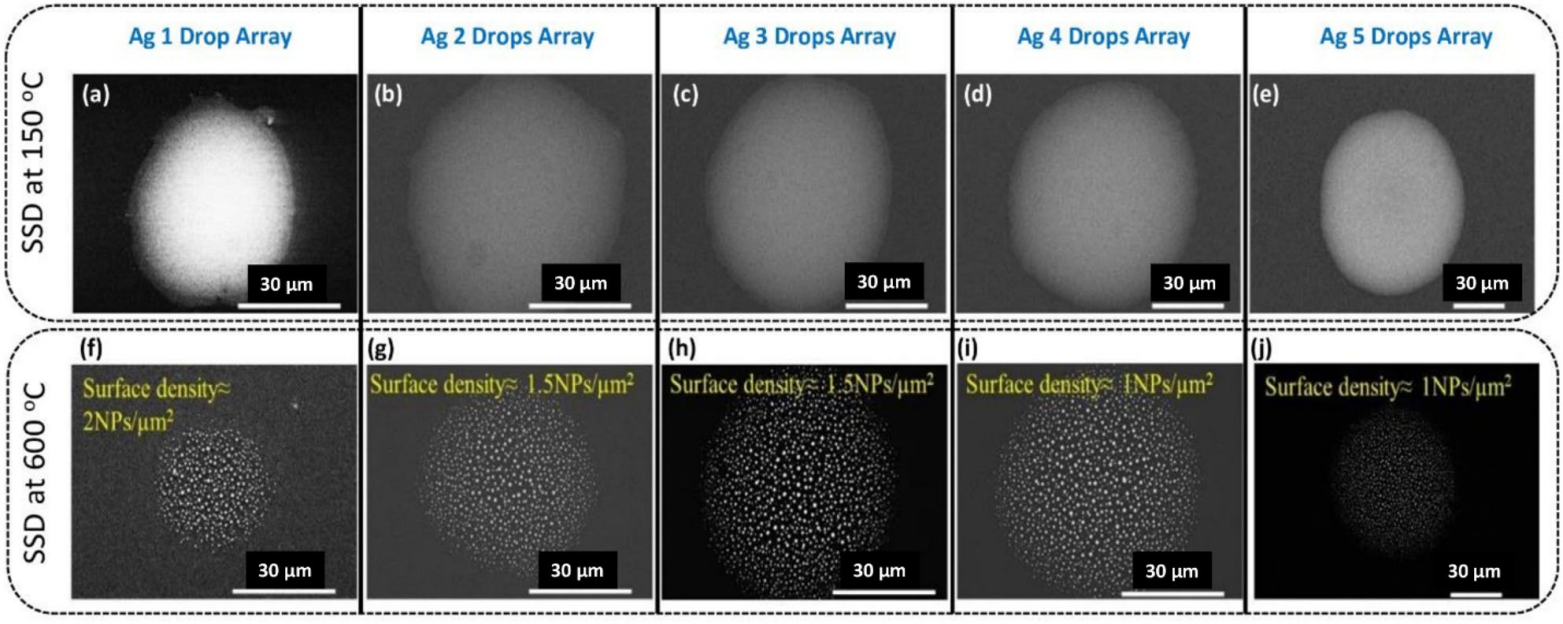
FESEM images of (1–5) drops Array of Ag µ-dots cured at (**a**–**e**) 50 °C for 30 min and (**f**–**j**) associated Ag nanoparticles chunks formation after SSD at 600 °C.

**Figure 6:**
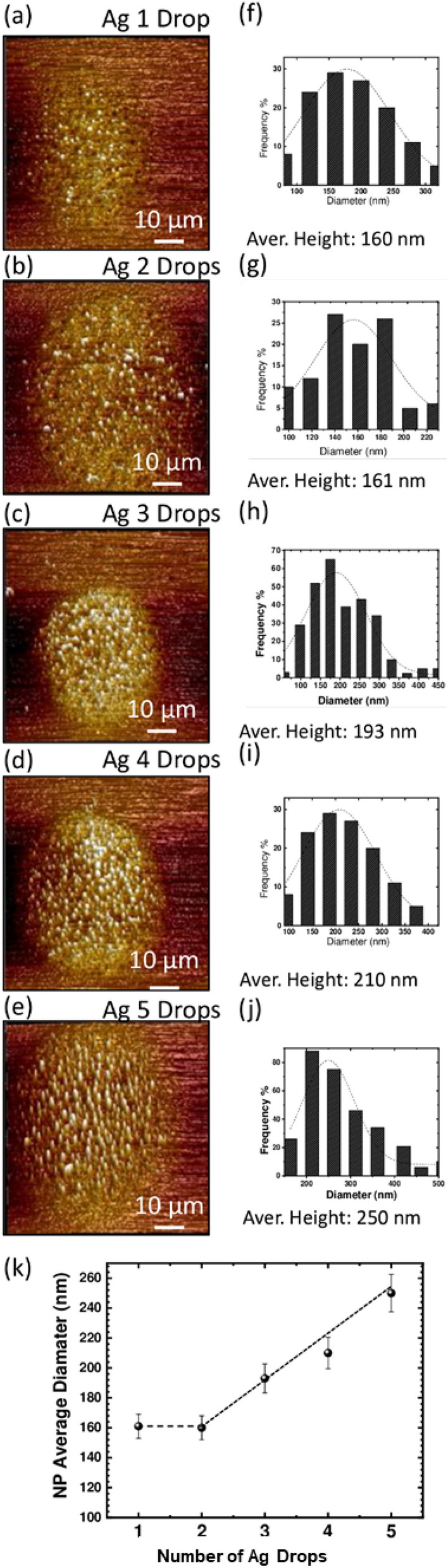
Atomic force microscopy analysis of Ag drops after Solid-State Dewetting (SSD) at 600 °C: (**a**) Ag 1 Drop, (**b**) Ag 2 Drops, (**c**) Ag 3 Drops, (**d**) Ag 4 Drops, (**e**) Ag 5 Drops. (**f**–**j**) Corresponding histograms depicting the size distribution of the average nanoparticle (NPs) diameters. (**k**) Plot illustrating the average diameter of the formed Ag NPs relative to the number of Ag drops.

Figure 4 illustrates the morphologies of the piezo inkjet-printed Ag µ-dots and the effect of Solid-State Dewetting (SSD) at 600 °C for 1 h. Figure 5 shows representative FESEM images of Ag µDots cured at 150 °C for 30 min and the subsequent formation of Ag nanoparticles chunks after SSD at 600 °C. Corresponding size distribution diagrams of the Ag µ-dots annealed at different temperatures are presented in Fig. 6. The size distribution plots reveal that dewetting at 600 °C for 1 h results in the formation of nanoparticles with varying sizes, ranging from less than 100 nm to up to 1000 nm. The EDS analysis of the SSD Ag µ-dots annealed at 600 °C is given in Figure S1(B). In all SSD Ag 1 to 5 µ-dots, a prominent Ag peak around 3 keV confirms the presence of silver. Notably, more than 90% of the dewetted nanoparticles are in the nanometer size range. Importantly, smaller nanoparticles are observed at the edges of the dewetted Ag µ-dots, attributed to the coffee ring effect, where Ag nanoparticles accumulate around the edges upon drying. It is well known that, in the coffee ring effect, the solvent evaporates faster at the edges, creating a flow from the droplet interior towards the outside.

**Figure 7:**
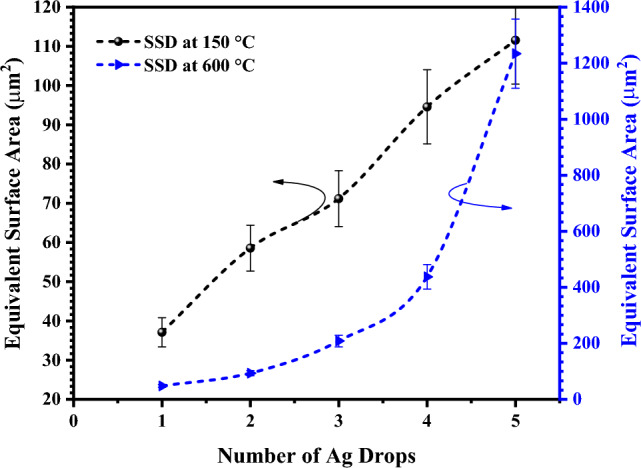
Comparison of “Equivalent Surface Area” of deposited Ag drops after a SSD at 150 °C and 600 °C.

The size distribution of Ag nanoparticles (AgNPs) in the dewetted drops occurring at 600 °C was calculated using ImageJ analysis. The particle size distribution, presented as histograms and average diameter for all Ag drop densities, is provided in Fig. 6. The histograms reveal that different NP diameters are formed from Ag 1 drop to 5 drops. To measure the aspect ratio of the dewetted Ag-µdots transformed into nanoparticles, scanning was performed using the Bruker ICOM Dimension AFM system. The system operates with a silicon tip in “ScanAsyst mode,” a combination of contact mode and tapping mode. A scanning force of 10 nN was utilized for imaging at a scanning rate of 0.5 Hz. Screenshots displaying the measuring parameters for AFM analysis are presented in Figure S2A, and Figure S2B showcases the AFM analysis of Ag microdots cured at 150 °C. AFM analysis of Ag microdots subjected to Solid-State Dewetting (SSD) and annealed at 600 °C for 1 h is illustrated in Fig. 7, with a more comprehensive and detailed analysis provided in Figure S3 (A to E).

ImageJ analysis was employed to measure the number of particles per unit area and the exposed specific surface area at both 150 °C and 600 °C, with corresponding values presented in Table 1. Following Solid-State Dewetting (SSD) at 600 °C, it was calculated that Ag 1 drop transformed into 802 AgNPs of varying diameters, while Ag 5 drops transformed into 4740 AgNPs of different diameters. Exposed specific surface area (SSA) was calculated for the printed Ag-µdots after curing at 150 °C, assuming each microdot to be a hemisphere. Similarly, the exposed SSA was calculated for the AgNPs obtained when the same printed Ag-µdots were transformed into equidistant nanoparticle chunks (i.e., after SSD at 600 °C). The exposed SSA was found to increase as the deposited ink varied from Ag 1 drop to Ag 5 drops. This increase in SSA has been illustrated in Fig. 7. Notably, the most significant increase in SSA was observed for an SSD of Ag 5 drops, with an associated value of 1006.13%. This substantial SSD value is attributed to the quantity of Ag nanoparticles present in 5 drops. The obtained SSA values for Ag 1 to 5 drops after SSD at 600 °C have been plotted and compared with SSA measured after curing at 150 °C, and the results are depicted in Fig. 7.

**Table 1:** Compared exposed specific surface area increase of Ag drops after SSD at 600 °C.

Parameters	Ag 1 Drop	Ag 2 Drops	Ag 3 Drops	Ag 4 Drops	Ag 5 Drops
µdots @150 °C	1	1	1	1	1
Particles @ 600 °C	802	2195	2514	3120	4740
SSA (µm^2^) @ 150 °C	37.11	58.57	71.17	94.56	111.54
SSA (µm^2^) @ 600 °C	47.87	93.58	208.47	437.50	1233.84
% increase in SSA	28.98	59.76	65.86	362.66	1006.13

**Figure 8:**
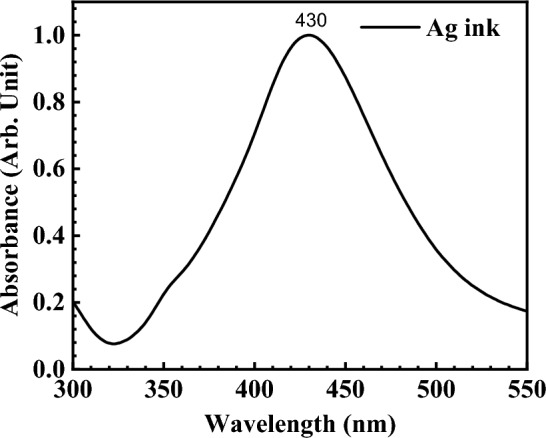
Absorption spectra of the Ag ink for inkjet printing.

The quality of the nanostructures resulting from the thermal treatment, particularly in relation to the presence of plasmon resonance, is manifested in the UV–Vis absorption spectra. The UV–Vis spectrum of Ag nanostructures is inherently complex, influenced by various factors such as the size, shape, changes in electronic structure, or the dielectric function of the medium in which these nanostructures are dispersed. Consequently, the absorption and scattering properties of Ag nanoparticles (AgNPs) can be finely tuned by controlling particle size, shape, and the local refractive index near the particle surface. For instance, spherical AgNPs typically exhibit a localized surface plasmon resonance (LSPR) absorption band around 400 nm. Therefore, the presence of a broad peak with a strong tail in the absorbance spectrum indicates a wide distribution of nanoparticle sizes^54^. In the case of spherical shapes where the diameter (D) is much smaller than the wavelength (λ), the resonance reflects only the dipole mode of the collective oscillations of electrons^55,56^. The absorbance of the Ag nanoparticles dispersion is illustrated in Fig. 8.

**Figure 9:**
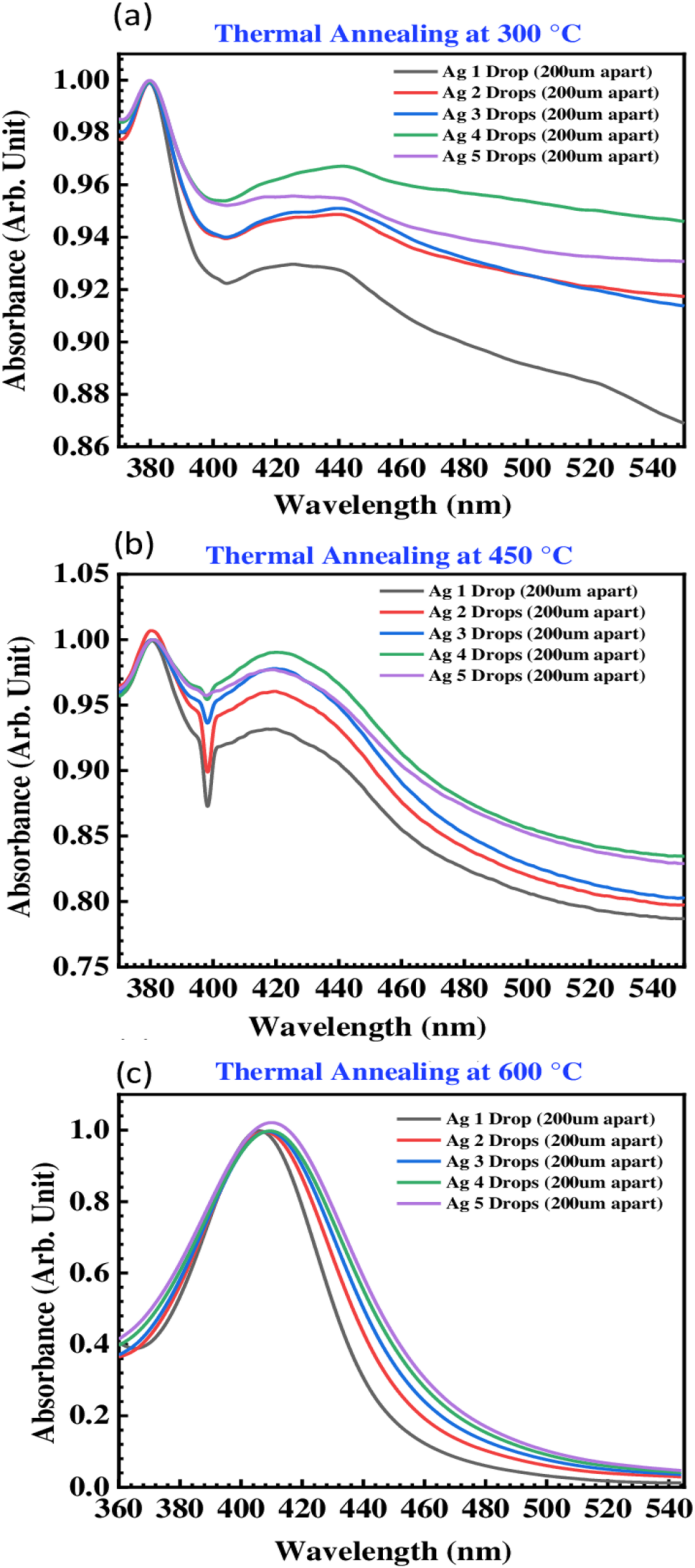
Absorption spectra of the printed Ag drops arrays printed at 200 µm apart while annealed at different temperatures: (**a**) 300 °C, (**b**) 450 °C and (**c**) 600 °C.

The absorbance of the inks for printing the Ag-µdots arrays (having a different initial thickness, and thermally annealed at 300 °C, 450 °C and 600 °C) is shown in Fig. 9. The red-shifted maximum absorption peak of printed Ag-µdots from 380 to 420 nm has attributed to the AgNPs formation. The tail associated with main absorption strong peak is due to the Ostwald ripening phenomenon^57^, based on which small particles lose stability and recombine into larger particles to achieve greater thermodynamic stability, causing a reduction in the surface-to-volume ratio^58^. As observed in FESEM and AFM analysis, all the five dewetted Ag-µdots (i.e., ranging from 1 to 5 drops) are containing different diameter nanoparticles distribution. AgNPs drops (1–5) arrays cured at 150 °C, has no absorbance peak. Similarly, arrays of AgNPs (1–5) drops annealed at 300 °C have shown a strong maximum absorption peak located at 380 nm when µdots printed 100 µm-apart, showing a broad peak around 440 nm and leaving a tail after, given in Figure S4 (A). It is observed that Ag-µdots printed 200 µm-apart, has been showing strong absorption peak at 380 nm, similarly, the printed 100 µm-apart Ag-µdots have a broad absorption peak around 440 nm with a tail leaving after. Absorption measured after SSD at 450 °C for 1 h is kept showing the peak at 380 nm, given in Figure S4 (B), and those annealed at 300 °C, have been showing a broad absorption peak at 420 nm and a leaving tail after this value. Interestingly, a sharp peak at 420 nm with dropping intensity at 400 nm appeared for all five Ag-µdots arrays. Absorption spectra measured for Ag-µdots annealed at 600 °C for 1 h, has been showing strong absorption peaks located at 410 nm for Ag 5 and 4 drops array, while for Ag 1 and 2 drops array, it is located at 406 nm and 407 nm, respectively. It is noteworthy at this point that in the absorbance spectra from Ag 1 drop to Ag 5 drops, the batches exhibit an increase in the number of larger diameter AgNPs within the respective chunks. Consequently, absorption peaks have been observed to initially broaden and then shift towards longer wavelengths. The optical properties of AgNPs have changed with the increasing number of larger diameter nanoparticles in the corresponding Ag microdrops.

It is evident that at 300 °C, Ag-µdots treated at both 100 µm and 200 µm-apart configurations exhibited a broad bump in the range of 400–500 nm, likely associated with the collective vibrations of free electrons (refer to Fig. 9). This bump becomes more pronounced and narrower when samples are annealed at 450 °C and 600 °C, as the conduction of electrons near each nanoparticle surface becomes delocalized and these electrons are also shared with neighboring particles. Consequently, the surface plasmon resonance shifts to lower energies, causing the absorption peaks to red-shift to longer wavelengths. One may observe a distinct decrease in absorbance near the 400 nm wavelength in Fig. 9b. While its origin remains unknown, its intensity appears to be inversely proportional to the number of Ag drops, speculatively suggesting a correlation with the amount of nanoparticles formed. In fact, with an increase in the number of Ag drops, there is a corresponding increase in absorption intensity. Additionally, it has been noted that as the temperature for Solid-State Dewetting (SSD) increases (i.e., from 300 to 600 °C), the absorption peak around the 400 nm wavelength becomes smoother.

Table 2 displays the change in the bandgap energy values of the Ag drops printed arrays when annealed at different temperatures. The peak at the wavelengths in the range 350–360 nm could correspond to quadrupole resonance, which is usually observed for nanostructures of larger size^59–62^.

**Table 2:** Change in bandgap energy of Ag drops printed arrays when annealed at different temperatures.

No. of Ag drops	100 µm apart Annealed at 300 °C Bandgap Energy (eV)	200 µm apart Annealed at 300 °C Bandgap Energy (eV)	200 µm apart Annealed at 450 °C Bandgap Energy (eV)			200 µm apart Annealed at 600 °C Bandgap Energy (eV)
1 Drop	3.27	3.27	3.27	2.95	3.11	3.06
2 Drops	3.26	3.26	3.26	2.95	3.11	3.06
3 Drops	3.26	3.26	3.26	2.95	3.11	3.03
4 Drops	3.26	3.26	3.26	2.95	3.11	3.02
5 Drops	3.26	3.26	3.26	2.95	3.11	3.02

The band gap of silver nanoparticles has been calculated from absorption spectra via Einstein Photon Energy relation:

1 $$E_{g} = \frac{hc}{{\lambda_{max} }}$$

2 $$E_{g} = \frac{1240}{\lambda } \left( {{\text{eV}}} \right)$$

The change in the band gap energy is due to the relationship between the optical absorption spectrum of metal nanoparticles caused by surface plasmon absorption and their sizes. The surface plasmon resonance is the *coherent excitation* of all the free electrons within the conduction band. Gustav Mie^63^ introduced the optical absorption of metal nanoparticles as LSPR. The absorption of light in metal nanoparticles can be described as intra-band excitations of conduction electron from the lowest energy state to higher energy states near the Fermi level of the conduction band upon receiving photon energy having the maximum absorbance wavelengths (λ_max_). Smaller particle sizes contains fewer numbers of atoms and reduces the potential attraction between the conduction electrons and metal ions of the particles. Due to this phenomenon, the conduction band energy increases for the smaller particles. However, for larger particle size which contains a large number of atoms, this is increasing the potential attraction between conduction electrons and metal ions and therefore reduces the conduction band energy of the metal nanoparticles^64–69^.

It has been observed that there is no change in the bandgap energies of the printed Ag microdots (1–5) arrays with 100 µm and 200 µm periods when annealed at 300 °C and 450 °C. However, when annealed at 600 °C, a slight change in bandgap energy of 0.04 eV has been noted, transitioning from 3.06 eV for Ag 1 drop to 3.02 eV for Ag 5 drops. This change can be attributed to the presence of Ag nanoparticles in the annealed microdots. As the number of deposited drops increases, the amount of Ag nanoparticles also increases. This rise in the number of Ag nanoparticles evidently results in a higher number of electrons, thus contributing to a slight reduction in the bandgap energy.

## Conclusions

In summary, we have demonstrated the utilization of a Piezo Inkjet printer with optimized printing parameters to achieve controlled printing of different arrays of Ag microdots (Ag-µdots) on a glass substrate by manipulating the number of Ag ink drops. These drops were deposited at the same position but with varying distances between consecutive printed dots (i.e., 100 µm and 200 µm). The printed Ag-µdots were cured at 150 °C for 30 min and subsequently annealed (Solid State Dewetting, SSD) at different temperatures: 300 °C, 450 °C, and 600 °C, for 1 h. Through FESEM and AFM analysis, it was observed that the printed Ag-µdots transformed into Ag nanoparticles (AgNPs) in chunks due to solid-state dewetting of the µdots after annealing at 600 °C for 1 h. This study represents the first of its kind, demonstrating the formation of equidistant chunks of AgNPs. UV–Vis analysis was conducted for both the as-printed Ag-µdots and those heat-treated at different temperatures. It was observed that, concerning the annealing temperature, the absorbance peaks of the Ag red-shifted, accompanied by a broadened tail, attributed to the formation of equidistant nanoparticle chunks composed of different sizes of Ag nanoparticles. This shift is attributed to the change in the exposed specific surface area after the formation of equidistant AgNPs chunks. This work can be extended to the formation of other metallic nanoparticles and even nanocomposites in chunks, providing a selective decoration of surfaces for specific applications.

### Supplementary Information

Supplementary Information.

## Data Availability

The datasets used and/or analyzed during the current study available from the corresponding author on reasonable request.
