# Supplementary material for: Piezo inkjet formation of Ag nanoparticles from microdots arrays for surface plasmonic resonance
Source: Sci Rep. 2024 Feb 27;14:4806. doi: 10.1038/s41598-024-55188-1 (PMC10899252; doi:10.1038/s41598-024-55188-1)
Supplement: Supplementary file 1 — Supplementary Information. [file 41598_2024_55188_MOESM1_ESM.doc]

**Supplementary Information**

**Piezo-Inkjet Formation of Ag Nanoparticles From Microdots Arrays For Surface-Plasmonic-Resonance**

Brahim Aïssa^1*^ and Adnan Ali^2^

*^1^Qatar Environment and Energy Research Institute (QEERI), Hamad Bin Khalifa University (HBKU), Qatar Foundation, Doha, P.O. Box 34110, Qatar*

*^2^Department of Chemical Engineering, Jeju National University, Jeju 63243, Korea*

********Correspondence: Corresponding authors*: Prof. B. Aïssa.

E-mail: [baissa@hbku.edu.qa](mailto:baissa@hbku.edu.qa)


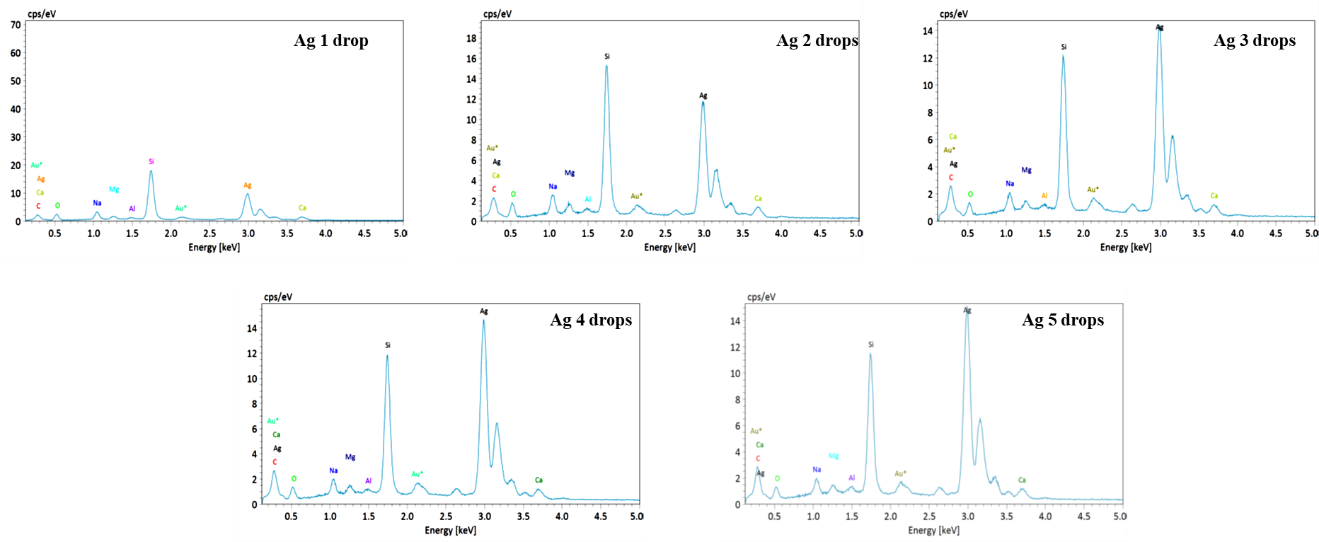


**A)**


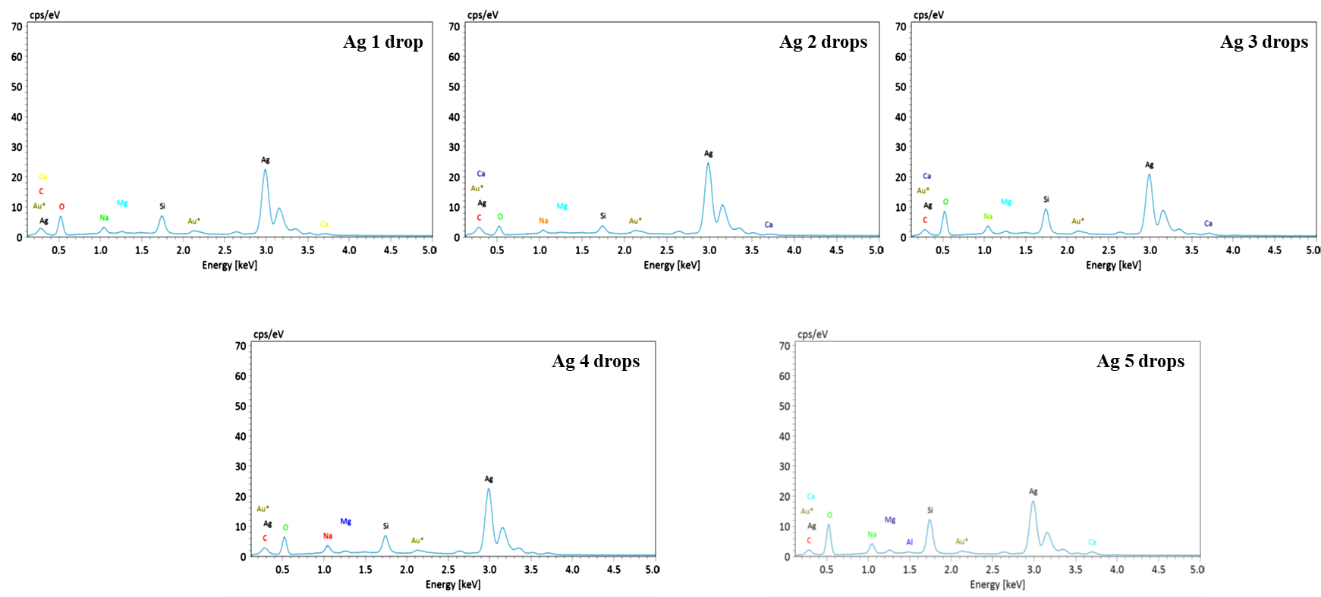


**B)**

**Figure S1:** EDS analysis of the Ag µ-dots after curing at A) 150 °C and B) 600 °C.

**
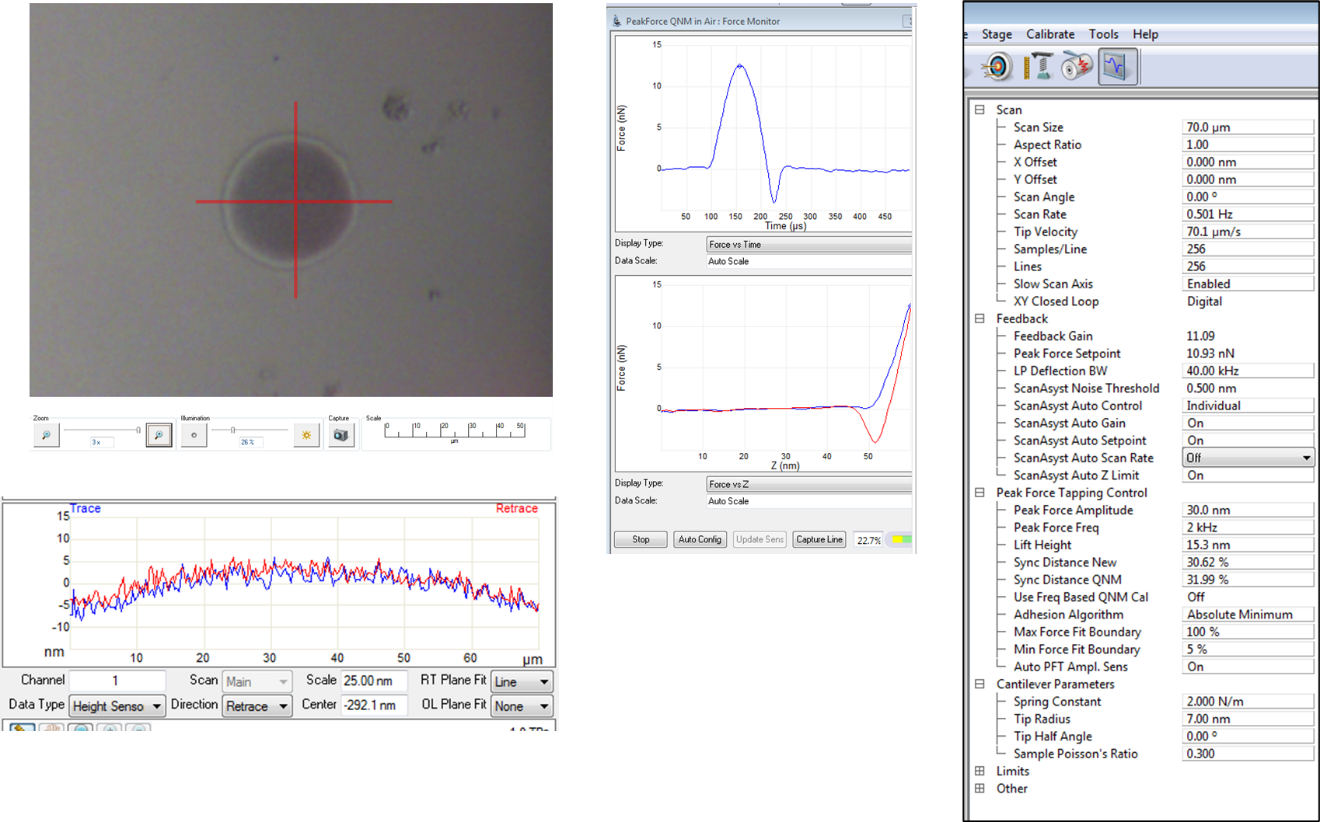
**

**A)**


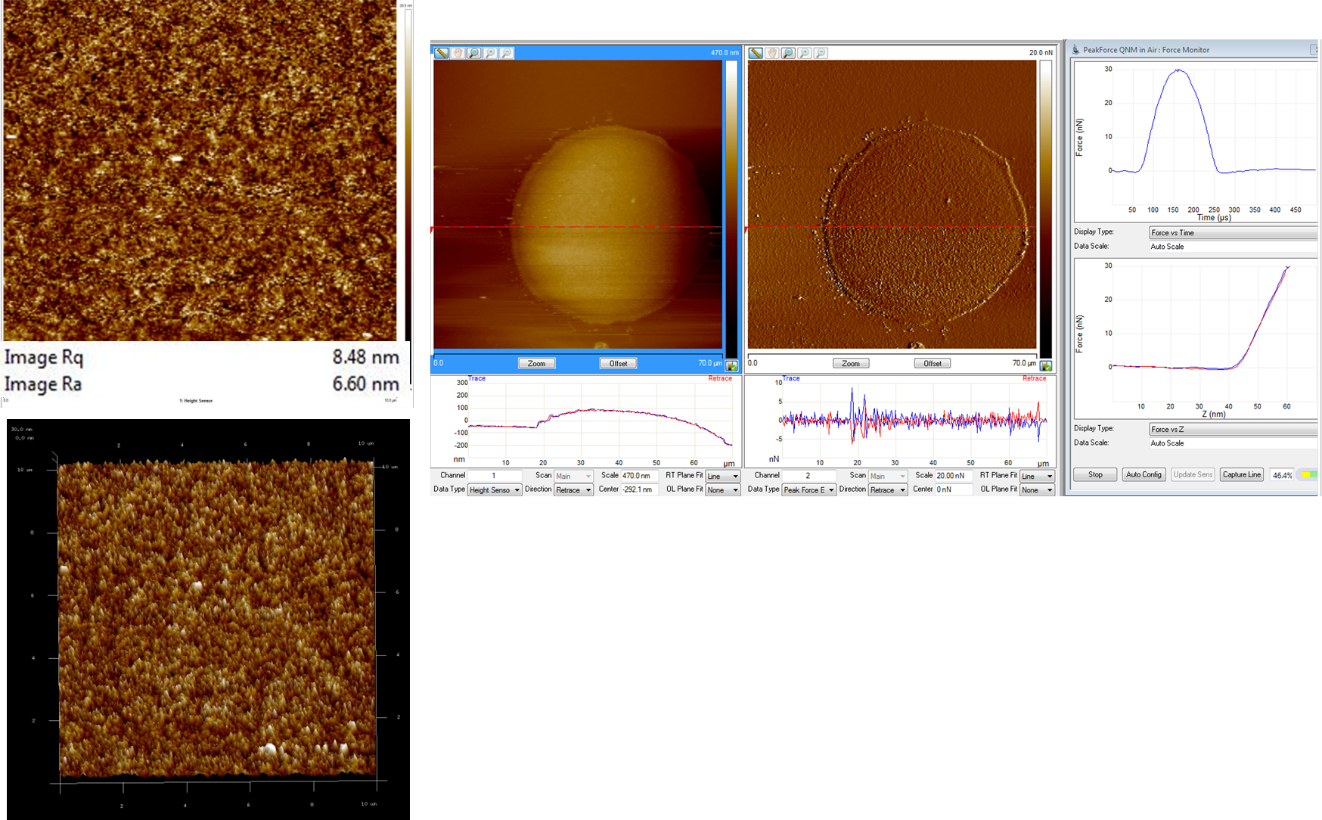


**Figure S2:** A) Measuring parameters for AFM Analysis of Ag microdots at different annealing temperatures, B) AFM Analysis of Ag printed drops cured at 150°C


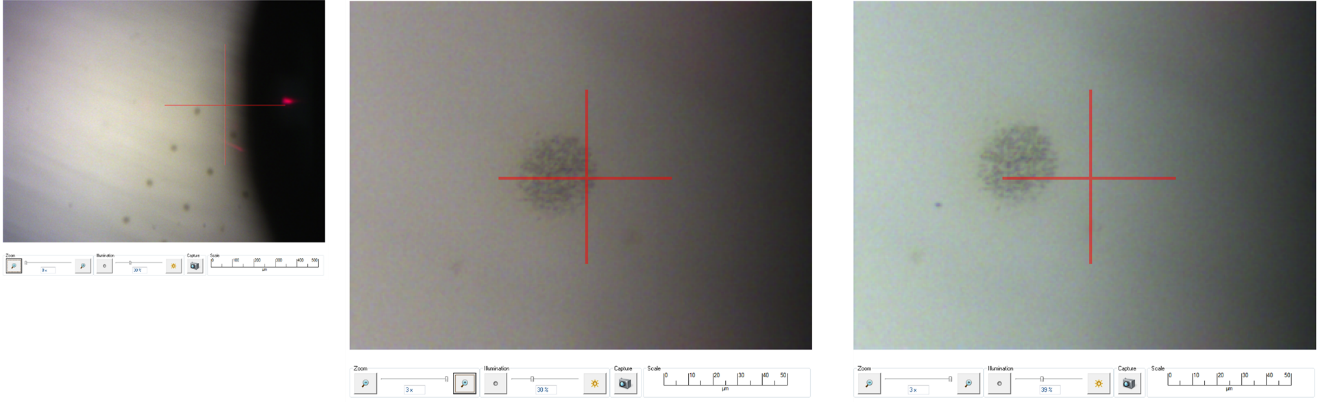


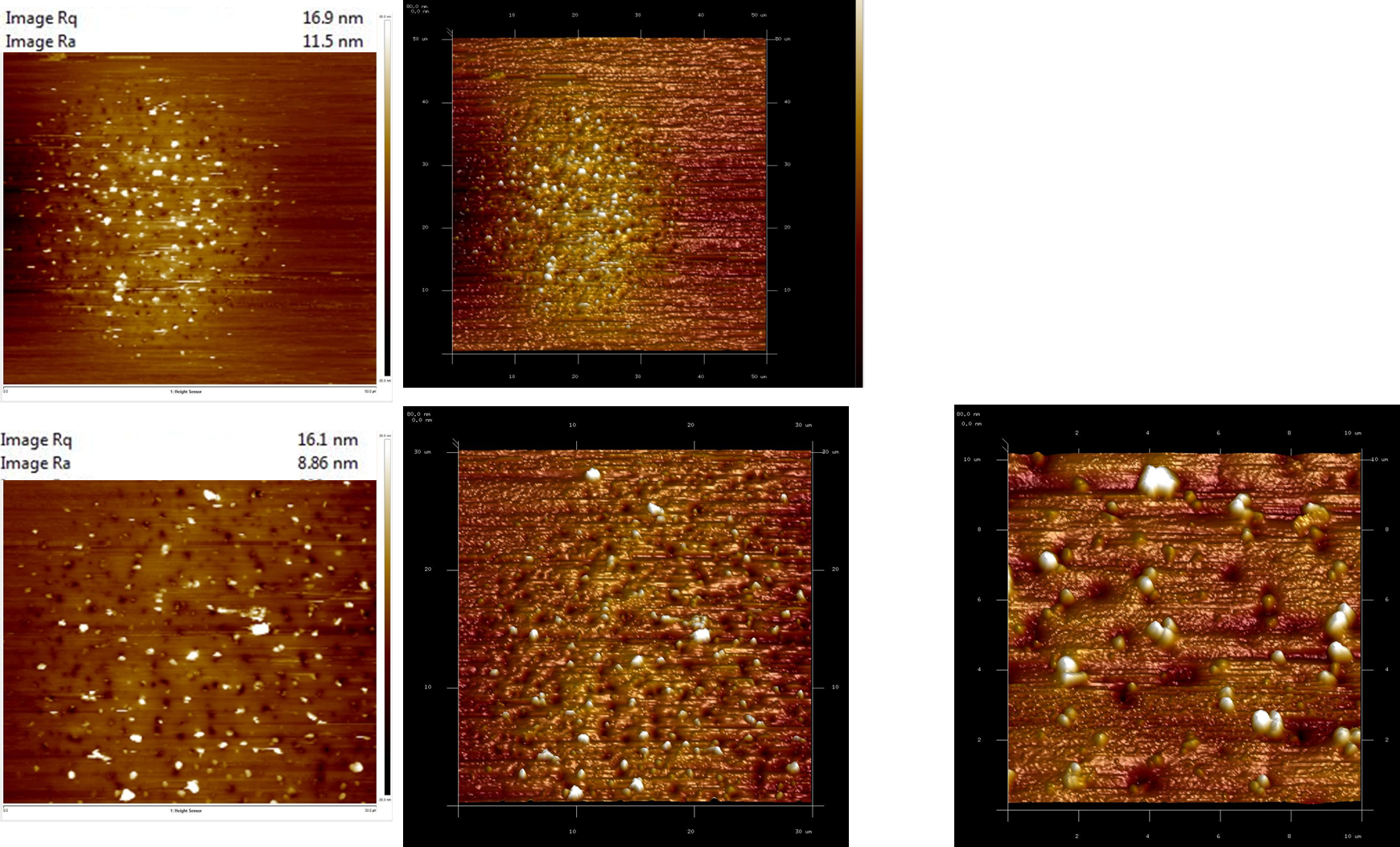


A) Ag 1 drop


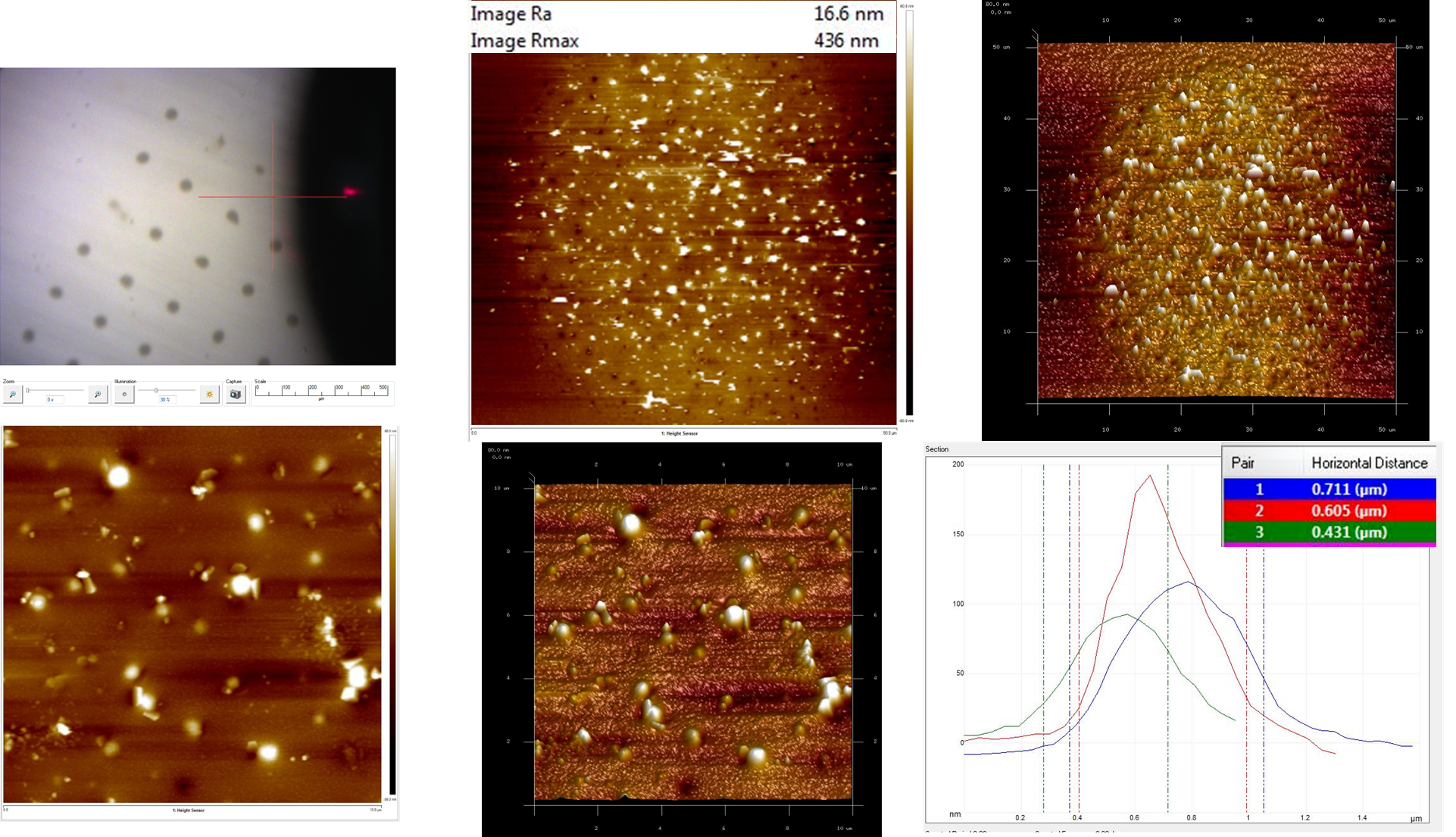


B) Ag 2 drops


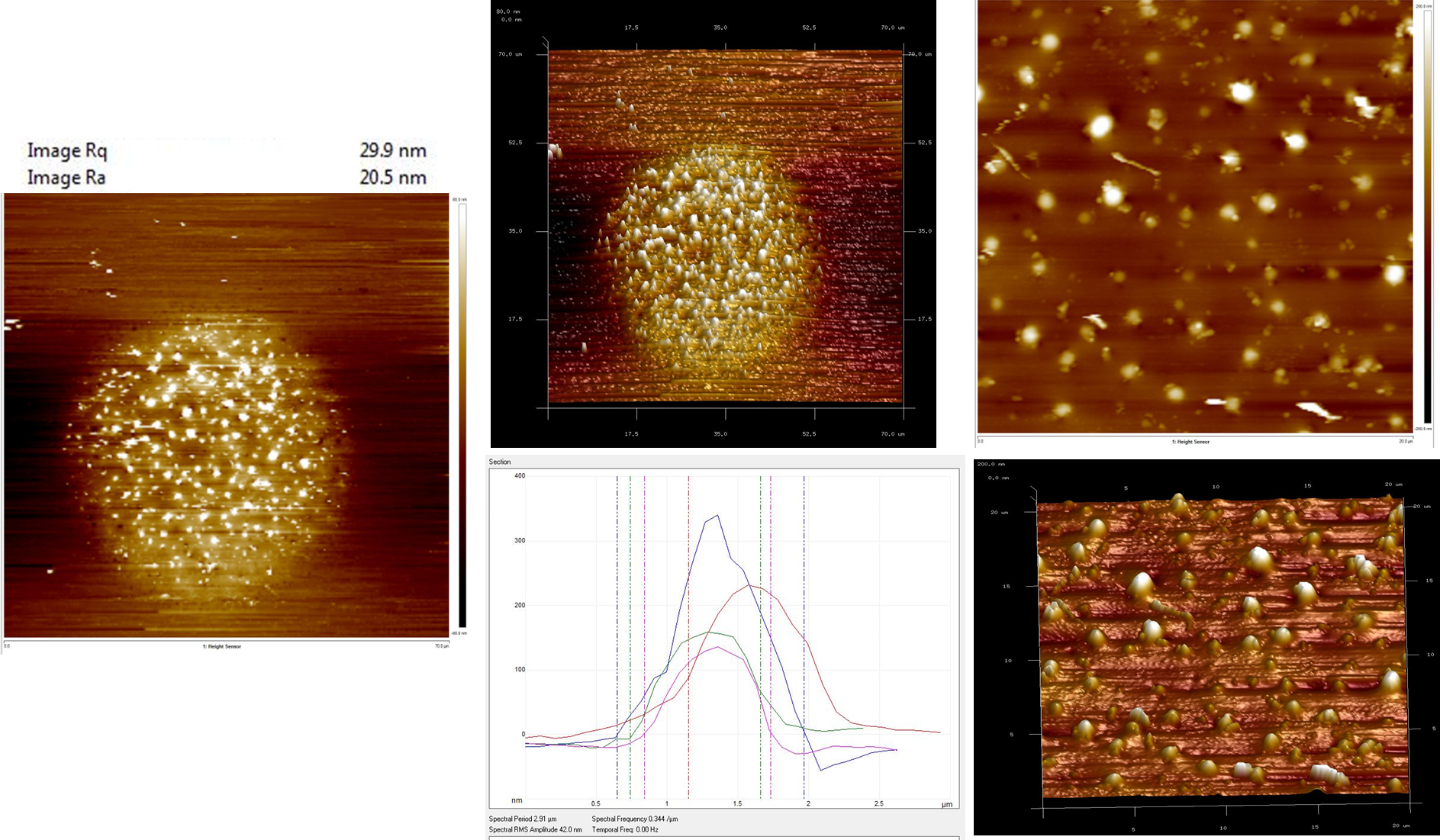


C) Ag 3 drops


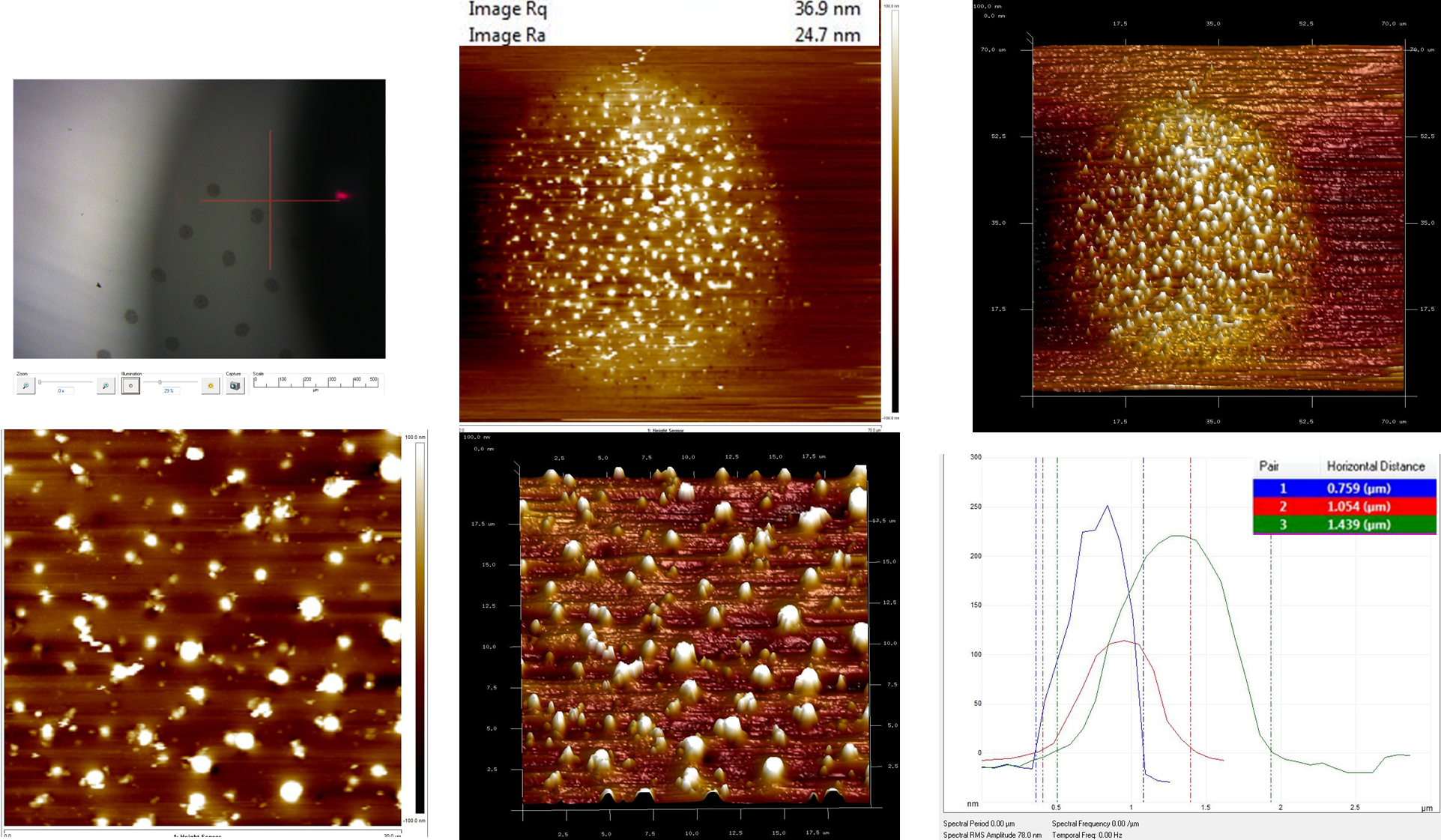


D) Ag 4 drops


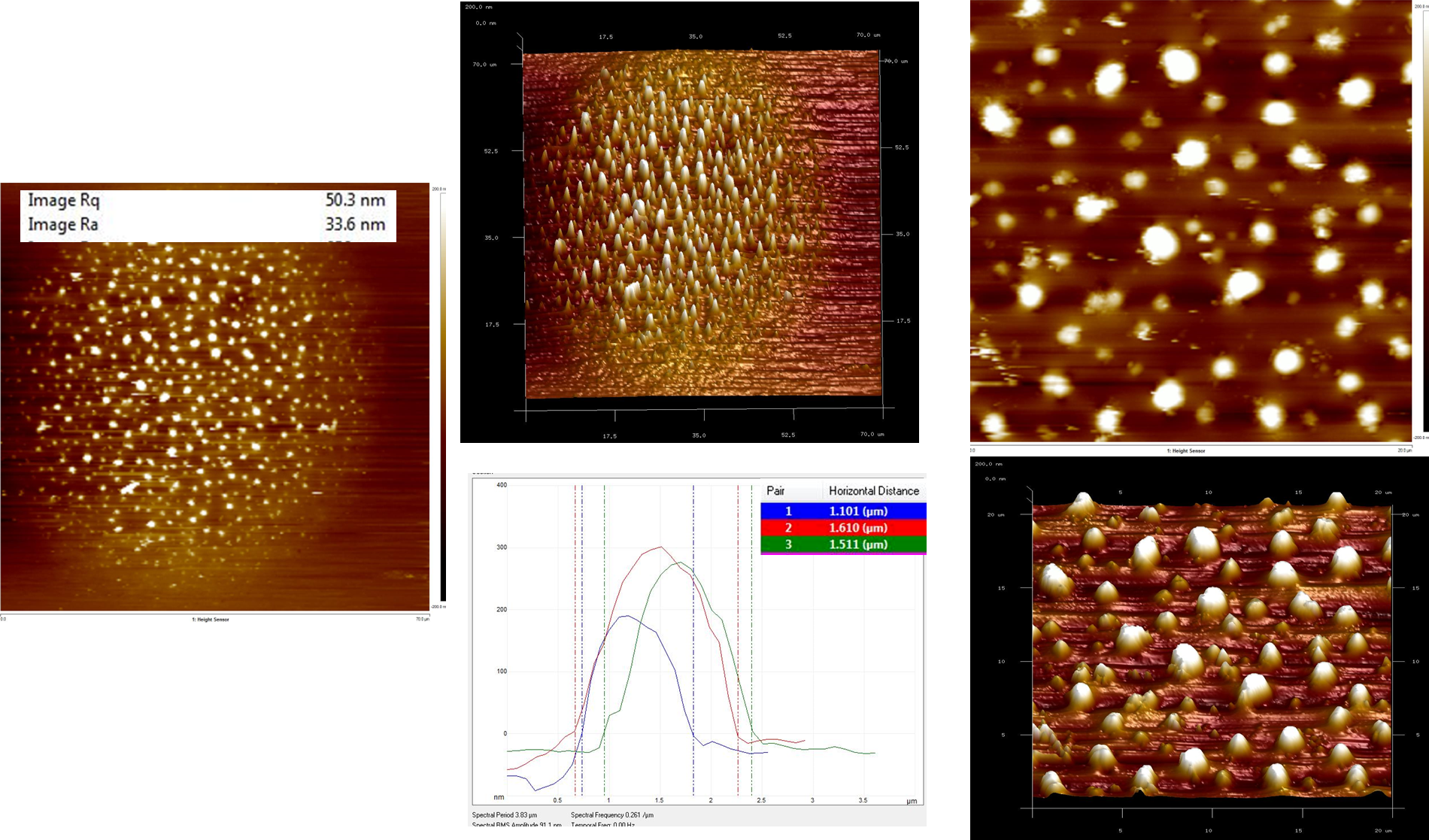


E) Ag 5 drops

**Figure S3:** A to E) AFM Analysis of SSD Ag 1 to 5 microdots annealed at 600°C.


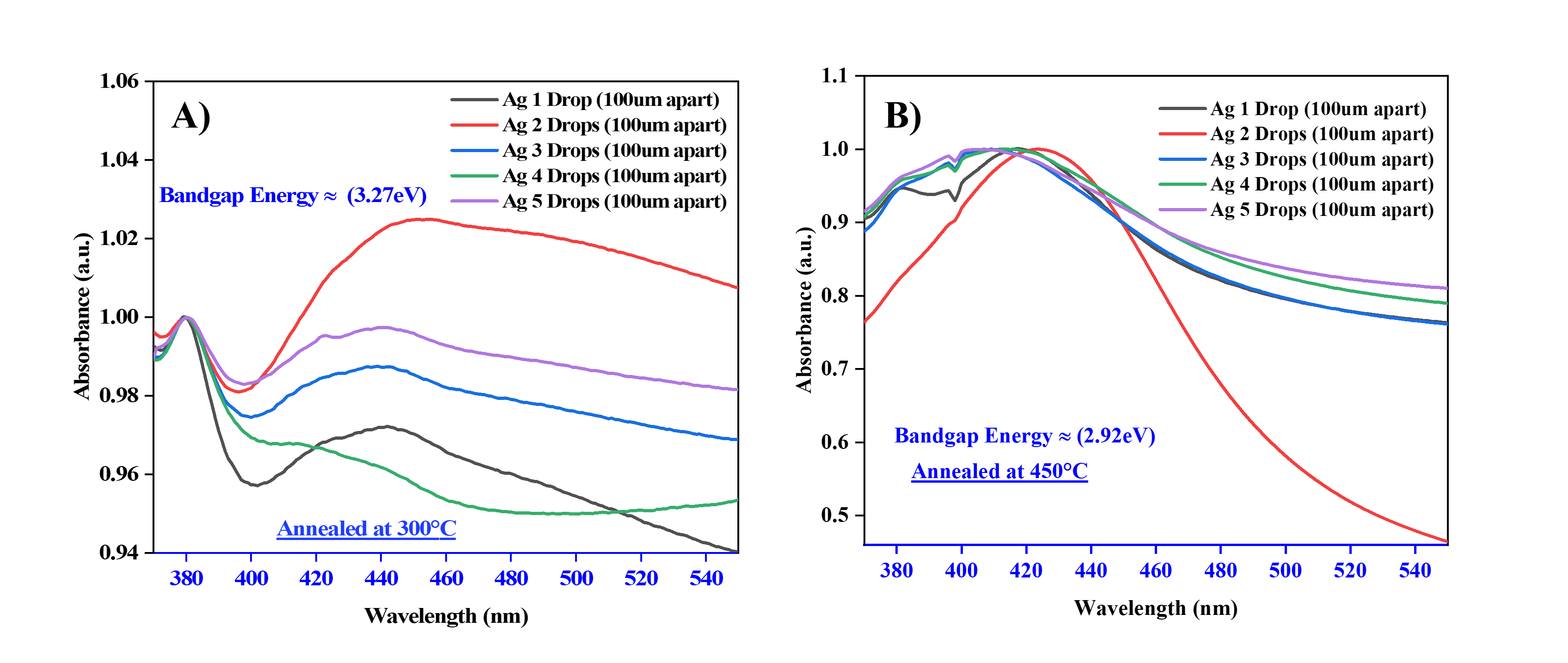


**Figure S4:** Absorption spectra of the printed Ag drops arrays printed at 100 µm apart annealed at 300°C and 450°C.
